# Continuous, Lateralized Auditory Stimulation Biases Visual Spatial Processing

**DOI:** 10.3389/fpsyg.2020.01183

**Published:** 2020-06-12

**Authors:** Ulrich Pomper, Rebecca Schmid, Ulrich Ansorge

**Affiliations:** ^1^Department of Cognition, Emotion, and Methods in Psychology, Faculty of Psychology, University of Vienna, Vienna, Austria; ^2^Cognitive Science Hub, University of Vienna, Vienna, Austria

**Keywords:** multisensory processing, dual-task, attention, cross-modal, response time

## Abstract

Sounds in our environment can easily capture human visual attention. Previous studies have investigated the impact of spatially localized, brief sounds on concurrent visuospatial attention. However, little is known on how the presence of a continuous, lateralized auditory stimulus (e.g., a person talking next to you while driving a car) impacts visual spatial attention (e.g., detection of critical events in traffic). In two experiments, we investigated whether a continuous auditory stream presented from one side biases visual spatial attention toward that side. Participants had to either passively or actively listen to sounds of various semantic complexities (tone pips, spoken digits, and a spoken story) while performing a visual target discrimination task. During both passive and active listening, we observed faster response times to visual targets presented spatially close to the relevant auditory stream. Additionally, we found that higher levels of semantic complexity of the presented sounds led to reduced visual discrimination sensitivity, but only during active listening to the sounds. We provide important novel results by showing that the presence of a continuous, ongoing auditory stimulus can impact visual processing, even when the sounds are not endogenously attended to. Together, our findings demonstrate the implications of ongoing sounds on visual processing in everyday scenarios such as moving about in traffic.

## Introduction

In natural environments, critical visual events regularly occur independent of or at different spatial locations than ongoing auditory stimuli. In traffic, for example, we have to monitor our visual environment simultaneously to hearing noise from nearby construction works, music being played on a car radio, or listening to a person next to us (cf. Cohen and Graham, 2003; Levy et al., 2006). Furthermore, as in these examples, sounds often are not transient events but continuous, ongoing streams. Thus far, little is known on whether such continuous auditory stimuli can attract human visual spatial attention. Moreover, it is unknown whether sounds need to be endogenously attended to, or whether the mere presence of a sound source might be sufficient for eliciting such a cross-modal spatial bias.

A multitude of previous studies have investigated the phenomenon of cross-modal spatial attention, in which orienting attention to a location in one modality (most commonly vision or audition) leads to a simultaneous shift of spatial attention in another modality (most commonly audition or vision, respectively; Spence and Driver, 1997; Driver and Spence, 1998; Eimer and Schröger, 1998; McDonald et al., 2000; Spence and Read, 2003). To a large degree, these studies have employed exogenous or bottom-up-driven shifts of attention. That is, spatial attention in one modality is automatically but only transiently shifted across modalities by a brief, salient cue from another modality. For instance, McDonald et al. (2000) had participants discriminate visual targets presented randomly in either the left or right periphery. Between 100 and 300 ms prior to the visual targets, a salient, spatially non-predictive auditory cue was briefly presented via a speaker from either the left or right side. The authors found that visual targets presented at the same spatial location as the preceding auditory cues were discriminated faster and more accurately than those presented on the opposite side, suggesting an automatic cross-modal shift of attention. Several other studies have demonstrated similar effects, both regarding behavioral reductions in response times (RTs) (McDonald and Ward, 2000; Kean and Crawford, 2008; Lee and Spence, 2015) and increases in performance accuracy (Dufour, 1999), as well as modulation of event-related potentials measured via electroencephalography (EEG; cf. Eimer and Schröger, 1998; Teder-Sälejärvi et al., 1999; Zhang et al., 2011).

In addition to such exogenous, bottom-up-driven effects, previous studies have also demonstrated instances of endogenous, top-down-driven cross-modal shifts of attention (Spence and Driver, 1996; Eimer and Driver, 2000; Green and McDonald, 2006). For example, Spence and Driver (1996) found links between endogenous auditory and visuospatial attention in an experiment, in which a central arrow cue indicated the likely location of a target stimulus in one modality. Occasional unexpected targets in the other modality were discriminated faster when appearing on the cued side rather than on the uncued side. This suggests that when participants deliberately direct their spatial attention to a location in one sensory modality, attention in a second modality shifts toward the same location.

However, although attention in the above example is not bottom-up driven, it still is shifted based on a discrete, brief onset of an external stimulus shortly before each visual target. This is clearly different from the everyday example described in the beginning, encompassing an ongoing auditory stimulus. A few studies (Santangelo et al., 2011, 2007) have demonstrated that endogenously attending to a visual or auditory rapid serial presentation (RSP) task can reduce or even eliminate exogenous spatial attention effects in the same or different sensory modality. In the experiment conducted by Santangelo et al. (2007), participants were presented with a peripheral spatial cueing task in either the auditory or visual modality. This task was presented either alone or simultaneously together with a centrally presented visual or auditory RSP task. The authors observed classical spatial cueing effects when the cueing task was presented in isolation, but no cueing effects when participants had to additionally perform the central RSP task. Interestingly, this was the case for both unimodal conditions (both cueing and RSP stimuli in the same modality) and cross-modal conditions (auditory cueing and visual RSP task, and vice versa). This experiment demonstrates that endogenously directing attention in one modality to a certain task and location affects exogenous attention in a different task at a different location. However, it still remains unclear whether this is due to a cross-modal shift in spatial attention or due to the endogenous attention task using up most of the limited attentional resources (cf. Kahneman, 1973; Lavie, 2005; Levy et al., 2006). Using an experimental design closer to everyday life, Driver and Spence (1994) demonstrated a cross-modal attentional bias from vision to audition. Here, participants were instructed to shadow one of two streams of speech, presented from equidistant locations in their right and left hemifields. Simultaneously, they had to monitor a quickly changing stream of unrelated visual stimuli for a target presented either from the same side or from the opposite side of the auditory target stream. Participants performed significantly worse in the speech-shadowing task when visual and auditory sources were presented from a different spatial location rather than the same spatial location, suggesting a cross-modal spatial bias from vision to audition. Interestingly, neither this (Driver and Spence, 1994) nor a later follow-up study investigating speech shadowing during a simulated driving task (Spence and Read, 2003) found evidence for cross-modal spatial bias in the opposite direction, from the auditory to visual domain. However, a further finding by Driver and Spence (1994) was that the auditory shadowing task was only affected when participants endogenously attended to the concurrent visual stream, but not when they merely viewed it passively.

Given evidence from dual-task studies, it is likely that not only active vs passive listening but also the level of semantic complexity, task difficulty, or load associated with the auditory task (Pomplun et al., 2001; Alais and Burr, 2004; Iordanescu et al., 2008, 2010; Mastroberardino et al., 2015) has an impact on the amount of cross-modal attentional bias. For example, Iordanescu et al. (2008) observed faster RTs in a visual search task when a sound associated with the target object was played during the search, even though it contained no spatial information (see also Van der Burg et al., 2008).

Taken together, previous work has shown that brief, salient auditory cues can attract exogenous visual attention. Endogenous cross-modal shifts of spatial attention using ongoing stimuli have so far only been demonstrated from the visual to auditory domain. In this case, only endogenously attending not passive viewing the visual stream has led to a spread of attention across modalities. To investigate the possibility of cross-modal shifts of attention from the auditory to visual domain, we presently asked the following three main questions: (1) To what extent can continuous, lateralized auditory stimuli bias visual spatial attention? (2) Is the mere presence of auditory stimuli sufficient to bias visual spatial attention, or do auditory stimuli need to be actively attended to in order to do so? (3) Does the semantic complexity of auditory stimuli impact their bias on visual attention?

In two experiments, participants discriminated visual targets in their left and right hemifields. At the same time, they had to either passively or actively listen to continuous lateralized auditory stimuli of varying semantic complexity. We measured the degree of attention directed to the visual targets through average correct RTs and accuracy of target discrimination at the same position as the auditory input (congruent condition) vs at a different position than the auditory input (incongruent condition). We show that even the mere presence of continuous auditory stimulation biases visual spatial processing and that, during active listening, overall visual task performance is dependent on the semantic complexity of the sounds.

## Experiment 1

### Methods

#### Participants

Twenty healthy university students participated in the experiment, in exchange for either course credits or monetary compensation. Our sample size was based on previous reports of cross-modal biases of attention, which commonly incorporated between 15 and 24 participants (e.g., Spence and Driver, 1996; Dufour, 1999; McDonald et al., 2000). Three datasets were excluded, as participants did not perform above chance level in the visual task. The remaining 17 participants (12 female; *M*_age_ = 25.6 years, range = 19 to 34) had normal or corrected-to-normal vision and were naive to the purpose of the experiment. All gave written informed consent, and the study was conducted in accordance with the standards of the Declaration of Helsinki. We further followed the Austrian Universities Act, 2002 (UG2002, Article 30 §1), which states that only medical universities or studies conducting applied medical research are required to obtain an additional approval by an ethics committee. Therefore, no additional ethical approval was required for our study.

#### Apparatus

Stimuli were presented on a 19-in. CRT monitor with a resolution of 1,024 by 768 pixels and a refresh rate of 85 Hz. Auditory stimuli were presented via two loudspeakers (Logitech Z150), placed directly left and right next to the monitor at the height of the visual targets and fixation cross. The distance between the centers of the two speaker membranes was 29.78° visual angle. Stimuli were controlled via an external USB sound card (Behringer U-Control UCA222). Sound levels were individually adjusted for each participant prior to the experiment, to be at a comfortable listening level [60–70 dB of sound pressure level (SPL); e.g., Andreou et al., 2015; Auksztulewicz et al., 2017; Barascud et al., 2016; Sohoglu and Chait, 2016]. Participants sat inside a dimly lit room 64 cm away from the screen, with their heads supported by a chin and forehead rest. The experiment was controlled by MATLAB (2014b v. 8.4.0, The MathWorks, Natick, MA, United States) using the Psychophysics Toolbox (Brainard, 1997) with the Eyelink extension (Cornelissen et al., 2002) on a PC running Windows 7.

#### Stimuli

Visual stimuli were presented against a black background (luminance: 8.2 cd/m^2^). Visual targets consisted of white triangles (17.5 cd/m^2^) presented at an eccentricity of 6.1° either left or right of a central fixation cross (0.4 × 0.4°). The triangles had an initial width of 0.7° and height of 0.4°. To titrate the average performance accuracy to around 75%, size and brightness of the triangles were increased or decreased by a factor of 0.05 after every fourth trial.

In the low-semantic complexity (LSC) condition, auditory stimuli consisted of a unilaterally presented stream of tone pips at a rate of 5 Hz. The individual tone pips had varying pitches within a frequency range of 310–1,000 Hz. The initial tone pip always had a pitch of 440 Hz, and the pitch for subsequent tone pips was randomly increased or decreased by 5%. In one third of the blocks, auditory stimuli were presented from the left speaker and in one third of blocks from the right speaker. As a baseline condition, no auditory stimulation was presented in the remaining third of the blocks. In the high-semantic complexity (HSC) condition, auditory stimuli consisted of a short story from Greek mythology (in German: *Inachos und Eris*; Köhlmeier, 2011). The story was taken from a publicly available online source. Again, the auditory stimuli were presented from the left and right speakers in one third of the blocks each. As a further baseline condition, auditory stimulation was provided bilaterally in the remaining third of the blocks.

#### Task and Procedures

**FIGURE 1:**
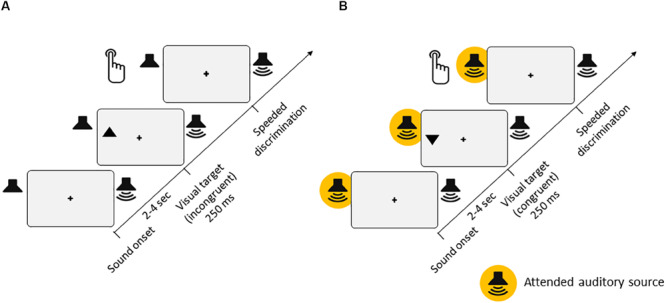
Experimental setup. **(A)** Experiment 1 [passive listening, for both low-semantic complexity (LSC) and high-semantic complexity (HSC) conditions]. Participants’ task was to discriminate the orientation of triangles (up or down) presented in either the left or right hemifield of the screen. On two thirds of trials, task-irrelevant auditory stimuli were presented from either a left or right speaker throughout each trial. In the LSC condition, the auditory stimuli consisted of a regular stream of tone pips with varying pitches. In the HSC condition, the auditory stimulus consisted of a spoken story. **(B)** Experiment 2 [active listening, for LSC, medium-semantic complexity (MSC), and HSC conditions]. The visual stimuli and task were identical to those of Experiment 1. Additionally, participants had to perform a parallel task in the auditory modality, which differed between the LSC, MSC, and HSC conditions (see section “Methods” for details).

The task was to discriminate the orientation of the target triangles (up or down) presented in either the left or right hemifield of the screen (Figure 1). A trial consisted of the presentation of a triangle for 250 ms, presented between 2 and 4 s after trial onset. For a triangle pointing upward, participants had to press Key *eight* with the right index finger on the number pad of the keyboard as fast as possible. Conversely, if the triangle was pointing downward, participants were instructed to press Key *two*. If no response was given within 1 s after onset of the visual target, the trial was counted as a *Miss*. During the trials, the index finger remained on the Key *five* to ensure that the distance to both response keys was equal.

In the LSC condition, participants completed six blocks with 42 trials each. Two thirds of trials contained unilateral auditory stimulation consisting of a continuous stream of tone pips, presented from the beginning of each trial. Blocks were alternating between left, right, and no auditory stimulations. In the HSC condition, participants completed six blocks of 34 trials each. Blocks were alternating between left, right, and bilateral auditory stimulations. Here, auditory stimulation consisted of an ongoing narrated story. Thus, in blocks with unilateral auditory stimulation, visual targets could be either spatially congruent (presented on the same side) or spatially incongruent (presented on the opposite side) with the auditory input. Importantly, the onset of visual targets was completely independent of (i.e., not time-locked to) the events in the auditory modality. In both conditions, participants were instructed to fixate the central cross throughout, focus on the visual task, and disregard the auditory input. Sound intensity was individually adjusted to each participant. Prior to the start of both complexity conditions, participants performed a short practice run to familiarize themselves with the task.

#### Eye Tracking

To ensure correct fixation throughout the experiment, gaze position at screen center was continuously ensured. Data were recorded monocularly using an EyeLink 1000 Desktop Mount (SR Research, Mississauga, Ontario, Canada) video-based eye tracker sampling at 1,000 Hz. Prior to the beginning of both conditions, the signal was calibrated on participants’ right eye using a nine-point calibration and validation sequence. Additionally, a recalibration of the eye tracker was performed in case participants left their position in the chinrest during breaks. An analysis and discussion of fixation behavior can be found in the Supplementary Information Figure S1.

#### Analysis of Behavioral Data

Prior to the statistical analysis, outlier trials with RTs deviating more than two *SD*s from the mean were excluded per participant and condition. Mean sensitivity (*d*′) and RT measures (for correct trials only) were computed separately for each condition. For the calculation of *d*′, we utilized the fact that our visual task required a discrimination between triangles pointing upward vs downward. We computed *d*′ separately for targets pointing upward and targets pointing downward and then averaged across them, according to the following equation:

$$d^{\prime }=\frac{z\,\left(p\,H\,i\,t_{u\,p}\right)-z\,\left(p\,F\,A_{u\,p}\right)+z\,\left(p\,H\,i\,t_{d\,o\,w\,n}\right)-z\,\left(p\,F\,A_{d\,o\,w\,n}\right)}{2}$$

Here, *Z* indicates a *z*-transform standardization. Trials in which participants gave an “upward” response by pressing Key *eight* were evaluated as hits, in case of triangles pointing upward (pHit_up_) and as false alarms, in case of triangles pointing downward (pFA_up_). Conversely, trials in which participants gave a “downward” response by pressing Key *two* were evaluated as hits, in case of triangles pointing downward (pHit_down_) and as false alarms, in case of triangles pointing upward (pFA_down_). For statistical analysis, sensitivity and RT data were each subjected to a repeated-measures analysis of variance (ANOVA), including the within-subjects variables Congruency (*congruent* vs *incongruent*) and Semantic Complexity (*low* vs *high*). To investigate potential differences between passive unilateral auditory stimulation (left or right) and no or bilateral auditory stimulation, we performed two additional repeated-measures ANOVAs. For the LSC only, we compared *congruent* vs *incongruent* vs *no-sound* trials. For the HSC only, we compared *congruent* vs *incongruent* vs *bilateral sound* trials.

## Results

**FIGURE 2:**
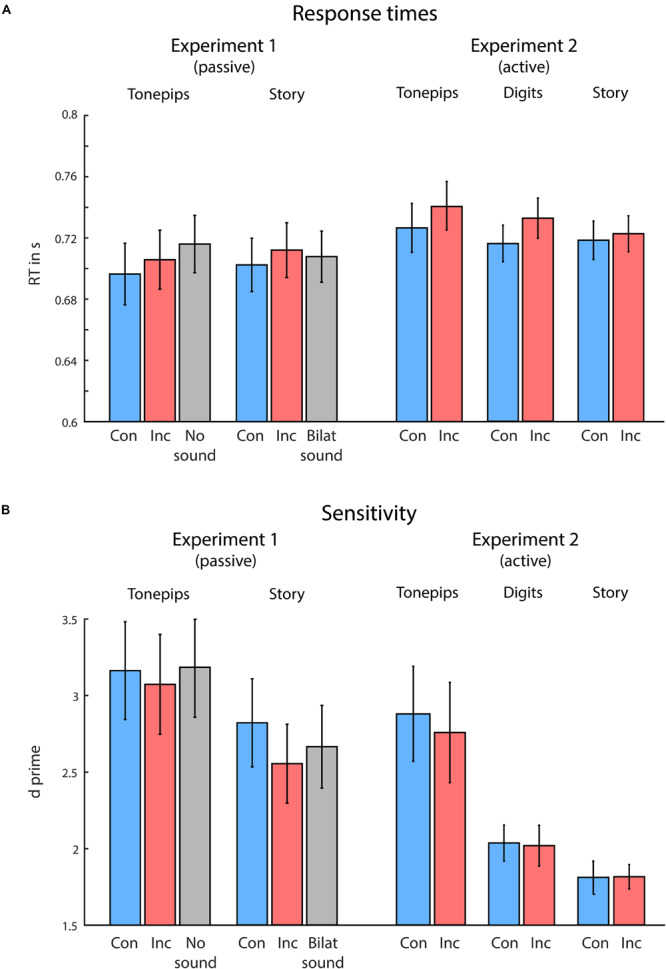
Behavioral results from Experiments 1 and 2, for the low-semantic complexity (Tone pips), medium-semantic complexity (Digits, only in Experiment 2), and high-semantic complexity (Story) conditions separately for congruent (blue) and incongruent (red) trials. **(A)** Visual response times (RTs). **(B)** Visual task sensitivity. Overall, congruency between visual and auditory spatial attention produced faster response times to visual targets, during both passive and active spatial listening conditions. Semantic complexity of the auditory stimuli modulated visual discrimination sensitivity, with higher semantic complexity leading to reduced sensitivity. Error bars indicate standard error of the mean.

Figure 2 illustrates the behavioral results. For both correct RTs and sensitivity, we performed a two-way ANOVA, using the variables Congruency (*congruent* vs *incongruent*) and Semantic Complexity (*low* vs *high*). For RTs, in line with our hypotheses, we found a significant main effect of Congruency, *F*(1,16) = 5.99, *p* = 0.026, *η*_p_^2^ = 0.27, owing to faster RTs in congruent compared with incongruent trials. Neither Semantic Complexity nor the interaction term reached significance (both *p*s > 0.740). Further, we observed no differences in sensitivity between conditions (all *p*s > 0.120).

We performed an additional ANOVA comparing the congruent, incongruent, and no-sound trials in the LSC condition (note that the no-sound condition was not included in the first ANOVA, as it was not present in the HSC condition). Here, we observed significant differences in RTs, *F*(1,15) = 5.0, *p* = 0.022, *η*_p_^2^ = 0.40, but not in sensitivity (*p* > 0.75). Follow-up *t*-tests (not corrected for multiple comparisons) yielded significantly faster RTs in the congruent compared with the no-sound condition, *t*(16) = −3.06, *p* = 0.007, *d* = 0.24, and in the incongruent compared with no-sound condition, *t*(16) = −2.51, *p* = 0.023, *d* = 0.13, but not in the congruent compared with incongruent condition (*p* = 0.115). Further, when comparing the congruent, incongruent, and bilateral-sound trials within the HSC condition (note that the bilateral-sound condition was not included in the above ANOVA, as it was not present in the LSC condition), we again observed significant differences in RTs, *F*(1, 15) = 4.43, *p* = 0.019, *η*_p_^2^ = 0.19, but not in sensitivity (*p* > 0.260). Follow-up *t*-tests (not corrected for multiple comparisons) yielded significantly faster RTs in the congruent compared with incongruent condition, *t*(16) = −2.67, *p* = 0.017, *d* = 0.13, but not in the congruent compared with bilateral-sound condition, or the incongruent compared with bilateral-sound condition (both *p* > 0.240).

### Discussion of Experiment 1

As hypothesized, we observed overall faster RTs to visual targets in congruent compared with incongruent trials. This is in line with previous studies showing both exogenous and endogenous cross-modal attentional biases from the auditory to the visual domain RTs (McDonald and Ward, 2000; Kean and Crawford, 2008; Lee and Spence, 2015). Importantly, however, participants in our experiment were instructed to ignore the auditory inputs and solely focus on the visual task. Thus, we demonstrate that the mere presence of an ongoing, task-irrelevant sound can affect concurrent visual spatial processing.

Further, we found neither a main effect nor an interaction including the variable Semantic Complexity. Presumably, when auditory input is not attended to, the nature or content of this auditory input is of little relevance for the overall visual task performance or the amount of cross-modal attentional spread. Importantly, however, whether or not an auditory stimulus was presented did affect task performance: Our participants were overall faster in trials featuring auditory stimulation (both congruent and incongruent) compared with the no-sound baseline trials (only present in the LSC condition). This suggests that ongoing sounds might increase the level of vigilance or overall, non-spatial attention, at least when listened to passively. Previously, such an effect has been shown for brief, task irrelevant sounds, which can transiently facilitate visual target detection (Kusnir et al., 2011). At the same time, further increasing the auditory input from one unilateral to two bilateral streams (only present in the HSC condition) did not result in any further significant reductions in RTs. We, thus, assume that the effect on vigilance due to the input of an additional sensory modality was already close to ceiling.

Overall, in Experiment 1, we demonstrate that the mere presence of a localized sound can spatially selectively bias performance in a visual attention task. In Experiment 2, we slightly modified our experimental paradigm to investigate how actively attending to a continuous lateralized auditory input would affect visual spatial attention. To induce the orienting of endogenous selective attention to one spatial location, we now presented two simultaneous continuous auditory streams, one from the left loudspeaker and one from the right loudspeaker. Here, participants had to selectively shift their auditory attention to one prespecified auditory stream while ignoring the other. Further, to investigate the potential impact of task difficulty more thoroughly, we added a third auditory stimulus condition of medium semantic complexity (MSC). Finally, owing to time limitations, we did not include a unimodal visual condition as in the LSC condition of Experiment 1.

## Experiment 2

### Methods

#### Participants

Twenty-two healthy university students participated in the experiment, in exchange for either course credits or monetary compensation. Three datasets were excluded, as participants did not perform above chance level in one of the auditory tasks. The remaining 19 participants (14 female, *M*_age_ = 23.0 years, range = 19 to 28 years) had normal or corrected-to-normal vision and were naive to the purpose of the experiment. All gave written informed consent, and the study was conducted in accordance with the standards of the Declaration of Helsinki.

#### Apparatus

The apparatus was identical to that of Experiment 1.

#### Stimuli

Visual stimuli were identical to those in Experiment 1. In the LSC condition, auditory stimuli consisted of bilaterally presented streams of tone pips at a rate of 5 and 6 Hz, respectively. In half of the blocks, the 5-Hz stream was presented from the right side and the 6-Hz stream from the left side, and vice versa. The individual tone pips had varying pitches within a frequency range of 310–1,000 Hz. The initial tone pip always had a pitch of 440 Hz, and the pitch for subsequent tone pips was randomly increased or decreased by 5%. In the MSC condition, auditory stimuli consisted of bilaterally presented, individual streams of digits (*0* to *9*, spoken in German). Digits were presented simultaneously from both sides at a rate of 1.5 Hz (mean digit duration = 500 ms, inter-stimulus interval, ISI = 166.6 ms). In the HSC condition, auditory stimulation consisted of two bilaterally presented short stories from Greek mythology. Both stories were told by the same narrator and taken from a publicly available online source. The target story was *Inachos und Eris* (Köhlmeier, 2011), and the distractor story was *Aigisthos* (Köhlmeier, 2013), both in German. The side from which the target story was presented alternated blockwise between the left and right speakers.

#### Task and Procedures

The visual task was identical to that of Experiment 1 (Figure 1B). Participants completed eight blocks with 12 visual targets each in the LSC and MSC conditions and six blocks with 34 trials each in the HSC condition. In addition to the visual task, participants had to perform a simultaneous auditory task. Prior to each block in all three conditions, they were instructed to direct their auditory attention to either the left or right speaker and to only listen to the respective auditory stream. The side to which they had to direct their auditory attention alternated between blocks.

In the LSC condition and MSC conditions, between zero and three auditory targets were presented in both the attended and non-attended streams. Auditory targets in the LSC condition consisted of an omission of one tone pip—that is, a gap in the stream. Auditory targets in the MSC condition consisted of a repetition of one digit, essentially constituting a one-back task. There were at least 10 tone pips or four-digit distance between two auditory targets, regardless from which side they were presented, and a minimum interval of 1,500 ms between visual and auditory targets. The laterality of auditory targets had no predictive power for subsequent visual targets. The participants’ auditory task was to count the targets only from the attended-to side. After each block, participants were asked to enter the number of detected targets via the keyboard. In both the LSC and MSC conditions, participants were explicitly allowed to keep track of the counted targets using their left hand, as we intended this to be an attention but not a working memory task.

In the HSC condition, participants were instructed to attentively listen to one of the simultaneously presented stories. The side from which the target story was presented and to which the participants had to listen to alternated between blocks. Between the blocks, participants had to complete a comprehension questionnaire of three to five questions about the content of the story in the previous block. This was to ensure that participants had permanently turned their auditory attention to the correct loudspeaker according to the instruction. Thus, similar to Experiment 1, visual targets were either spatially congruent or incongruent with the current locus of auditory attention. Sound intensity was individually adjusted for each participant as in Experiment 1. Prior to the start of each condition, participants performed a short practice run to familiarize themselves with the task.

#### Eye Tracking

Eye tracking was performed in the same way as in Experiment 1. An analysis and discussion of fixation behavior can be found in the Supplementary Information Figure S2.

#### Analysis of Behavioral Data

Prior to the statistical analysis, outlier trials with RTs deviating more than 2 *SD*s from the mean were excluded per participant and condition. Mean sensitivity (*d*′) and RT measures (for correct trials only) were computed separately for each condition. For statistical analysis, sensitivity and RT data were each subjected to a repeated-measures ANOVA, including the within-subjects variables Congruency (*congruent* vs *incongruent*) and Semantic Complexity (*low* vs *medium* vs *high*).

To investigate potential differences in RTs and sensitivity between passive and active listening conditions, we performed an additional mixed-model ANOVA, using Listening Condition (*Experiment 1—passive* vs *Experiment 2—active*) as a between-subjects variable and Congruency (*congruent* vs *incongruent*) and Semantic Complexity (*low* vs *high*) as within-subjects variables. Where appropriate, the reported results are corrected for violations of sphericity.

#### Analysis of Auditory Task Performance

We additionally analyzed auditory task performance to ensure that participants complied with the instructions and shifted their audio-spatial attention accordingly. For each block of the LSC and MSC conditions, we compared the reported number with the actual number of auditory targets. For the HSC condition, we simply counted the number of incorrect responses. Because the auditory tasks were challenging, and participants on average gave incorrect responses to 28.7% (SEM: 4.1%) of auditory targets, excluding each block with an incorrect response or every participant that made a mistake on more than half of the blocks would have resulted in a too significant data loss. Thus, for each condition separately, we excluded subjects who made more than one error in more than half of the experimental blocks. Using this criterion, we excluded two participants on the basis of their performance in the LSC and one participant on the basis of the performance in the HSC condition.

## Results

Figure 2 illustrates the behavioral results. For both RTs and sensitivity, we performed a two-way ANOVA, using the variables Congruency (*congruent* vs *incongruent*) and Semantic Complexity (*low* vs *medium* vs *high*). For RTs, in line with our hypotheses, we found a significant main effect of Congruency, *F*(1,18) = 18.17, *p* < 0.001, *η*_p_^2^ = 0.50, owing to faster RTs in congruent compared with incongruent trials. Neither Semantic Complexity nor the interaction term reached significance (both *p*s > 0.160). For sensitivity, we observed a significant effect of Semantic Complexity, *F*(1,36) = 14.89, *p* < 0.001, *η*_p_^2^ = 0.45. Neither Congruency nor the interaction term reached significance (both *p*s > 0.640). Follow-up *t*-tests revealed significant differences in sensitivity between each of the conditions, owing to higher sensitivity in the condition with lower semantic complexity [tone pip compared with digits condition: *t*(18) = 3.51, *p* < 0.003, *d* = 0.54; tone pip compared with story condition: *t*(18) = 4.29, *p* < 0.001, *d* = 0.68; digits compared with story condition: *t*(18) = 2.88, *p* = 0.009, *d* = 0.41].

Using a mixed-model ANOVA, we additionally compared the performance between Experiment 1 (passive listening) and Experiment 2 (active listening). Again, in line with our hypotheses, we found an overall main effect of Congruency for RTs, *F*(1,34) = 13.30, *p* < 0.001, *η*_p_^2^ = 0.28, owing to faster RTs in the congruent compared with incongruent condition. We observed no additional effects on RTs (all other *p*s > 0.261). For sensitivity, we found an effect of Semantic Complexity, *F*(1,34) = 16.09, *p* < 0.001, *η*_p_^2^ = 0.32, owing to higher sensitivity in the LSC compared with HSC condition. Furthermore, we observed a trend toward significance in the variable Listening Condition, *F*(1,34) = 3.58, *p* = 0.067, *η*_p_^2^ = 0.10, owing to higher sensitivity in the passive compared with active condition. No other effects were found (all other *p*s > 0.12).

### Discussion of Experiment 2

In line with our hypothesis and Experiment 1 results, we observed faster RTs to visual targets in congruent compared with incongruent trials. As participants now had to endogenously and selectively direct their auditory attention to one side while ignoring the other, this setup is more similar to previous reports of endogenous cross-modal attention featuring continuous auditory stimulation (Driver and Spence, 1994; Spence and Read, 2003). The important difference is, however, that we here demonstrate an attentional bias from the auditory to the visual modality, which has not been shown before.

We expected that the strength of cross-modal bias would increase compared with the passive listening in Experiment 1, as participants now had to actively listen toward one auditory input. However, we observed no interaction between Experiments and Congruency effects. A possible conclusion is that, at least given our present setup, RTs in the visual task are affected independently of whether or not concurrent sounds are endogenously attended to. Alternatively, our mixed-model analysis might have been statistically underpowered to reveal a potential effect of active vs passive listening on the cross-modal attentional bias. This is supported by that fact that, here, unlike in the passive listening condition, semantic complexity of the sounds influenced visual target discrimination sensitivity. To help interpret this finding, we additionally calculated Bayes factor (BF; according to Rouder et al., 2012) for the interaction between Experiments and Congruency effects. We found a small BF for both RTs (BF = 0.24) and sensitivity (BF = 0.28), thus supporting the general lack of an effect.

We also found a trend toward significance in the comparison of sensitivity between the passive and active conditions. These observations speak against the conclusion that visual target discrimination performance was unaffected by whether the auditory input required active or passive listening. Overall, visual sensitivity parametrically decreased with increasing auditory semantic complexity, suggesting that the latter was associated with task difficulty or level of general, non-spatial distraction. Using non-spatial tasks, earlier studies have shown analogous effects of auditory task difficulty on the performance of parallel visual tasks (Pomplun et al., 2001). Thus, we here extended our findings from Experiment 1, in demonstrating that not only passive but also active spatial listening leads to a cross-modal shift in visual attention. At the same time, the overall performance in the parallel visual task is modulated by the semantic complexity of the attended auditory input.

## General Discussion

In the present study, we investigated exogenous and endogenous auditory-to-visual cross-modal shifts of attention using continuous streams of sound. With regard to our three main questions outlined in the beginning, we found that (1) continuous auditory inputs can facilitate the processing of spatially close visual stimuli; (2) not only actively listening to but even the mere presence of such auditory stimuli is sufficient to bias visual spatial processing; and (3) the semantic complexity of such auditory stimuli does impact the overall visual task performance but not in a spatially specific manner.

To the best of our knowledge, our study is the first to present evidence that attending to a continuous auditory stimulus biases visual spatial processing such that processing of visual stimuli close to the locus of auditory attention is facilitated. Previous work investigating this matter has demonstrated the reverse case of cross-modal attentional shifts from vision to audition (Driver and Spence, 1994; Spence and Read, 2003). For instance, the participants in the study by Spence and Read (2003) had to perform a simulated visual driving task. At the same time, they were instructed to shadow speech sounds presented either from the front (spatially congruent with the visual task) or from their side (incongruent with the visual task). The authors found facilitated shadowing performance when sounds were presented spatially congruent with the visual task but found no effect of sound location on visual task performance. As a potential reason for this, they suggested that participants likely prioritized the driving over the shadowing task, as the consequences of making a mistake in the former in real life are far more severe. The fact that we found an auditory to visual bias in the present study could be a consequence of the simpler visual discrimination task, which might be more susceptible to a cross-modal bias of attention. In this regard, our results are also in line with those of Santangelo et al. (2007), who demonstrated impaired peripheral visual cuing effects, when participants had to perform a simultaneous centrally presented auditory RSP task. Our study adds to these data by showing that the cross-modal effect is spatially selective—that is, responses to visual targets are facilitated if they appear spatially close to a continuous auditory input.

Crucially, we observed a cross-modal spatial facilitation not only in the active listening condition (Experiment 2) but also when participants were merely passively presented with a continuous unilateral auditory input, which they were instructed to ignore (Experiment 1). Thus, continuous auditory stimuli seem to inevitably attract attention to influence the processing of spatially close visual stimuli. Similar results were previously found in experiments using salient, sudden onset sounds, which also cause an automatic cross-modal shift in visual attention, even when sounds are task-irrelevant and participants try to ignore them (Dufour, 1999; McDonald and Ward, 2000; Spence and Read, 2003; Kean and Crawford, 2008; Lee and Spence, 2015). Yet this present result is somewhat different from what Driver and Spence (1994) reported in an endogenous, visual-to-auditory cross-modal attention paradigm. In their study, an auditory shadowing task was only affected when participants actively attended to a concurrent visual stream, but not when they viewed it passively. Although the results of their and our present study are difficult to compare owing to their use of largely different stimuli and tasks, this discrepancy might hint at an interesting asymmetry between the auditory and visual modality in their general ability to attract attention. In this line, audition has been suggested to serve as an “early-warning” system partly due to its ability to receive input from all spatial directions at all times (Dalton and Lavie, 2004; Murphy et al., 2013; Zhao et al., 2020) as well as its faster processing speed (Rolfs et al., 2005).

Although we expected that the strength of cross-modal attention effects would depend on whether sounds are task irrelevant or actively attended to, we only found a statistical trend toward that result. Using non-spatial dual-task paradigms, an earlier work has demonstrated a reduction in visual task performance during active compared with passive parallel auditory listening conditions (Kunar et al., 2008; Gherri and Eimer, 2011). It is possible that the additional demands of bilateral stimulation in our paradigm have reduced the observable cross-modal biasing effects, thus requiring a larger sample size to reveal performance differences between active and passive stimulation conditions. However, our BF analysis speaks against this possibility.

We did, however, find a modulation of visual task performance by semantic complexity in the active listening condition. Visual target discrimination sensitivity was the largest when the auditory stream consisted of tone pips (LSC), medium when it consisted of spoken digits (MSC), and smallest when it consisted of the narrated story (HSC). This finding fits well with typical previous results from dual-task studies, which demonstrated a reduction of performance in one task with increasing load or difficulty in the second task (Gherri and Eimer, 2011; Pizzighello and Bressan, 2008; Pomplun et al., 2001), as well as with studies showing the benefit of presenting semantically meaningful and target-related auditory cues along with a visual search task (Iordanescu et al., 2010, 2008; Mastroberardino et al., 2015). Interestingly, semantic complexity had only an overall effect on visual performance, and not on the amount of cross-modal attentional bias in our study. It is possible that semantic complexity has a stronger impact on the perceptual processing stage of visual input, causing the effects in discrimination sensitivity. At the same time, the general presence of a lateralized sound might impact the peri-perceptual stages (arousal, decision, and response speed) and, thus, affect visual RTs in a more cross-modal, spatially specific manner, similar to a phasic alerting signal (Coull et al., 2001; Sturm and Willmes, 2001; Fan et al., 2005; Haupt et al., 2018). The latter conclusion is further supported by our finding that RTs were slower in the no-sound compared with either the congruent or incongruent conditions, potentially driven by an increase in arousal leading up to faster visual processing (Qi et al., 2018).

A crucial aspect of our current design was the use of continuous auditory streams, which we believe to be fundamentally different from the brief, highly salient cues used in previous demonstrations of cross-modal shifts of attention (Spence and Driver, 1997; McDonald et al., 2000; Green and McDonald, 2006; Kean and Crawford, 2008). Still, one might argue that our presently used auditory stimuli are essentially just a series of individual, highly salient, abrupt-onset events drawing exogenous attention. However, we suggest this to be unlikely for two reasons. First, owing to the regularity and temporal predictability of the auditory input, at least for the conditions containing tone pips and digits, the individual sounds are arguably less salient and easier to ignore than are unpredictable, random-onset, brief auditory cues (Noyce and Sekuler, 2014; Havlíček et al., 2019). Second, we observed a similar, spatially specific visual processing bias in both the passive (Experiment 1) and active listening (Experiment 2) conditions. During the active condition, two separate auditory streams were presented continuously from the left- and right-sided speakers. Thus, auditory stimuli were presented from both sides also during visual target presentation. It follows that endogenous auditory attention would have been directed toward both sides similarly. However, we found a difference between attended and unattended side, making it highly unlikely that the facilitated processing of spatially congruent visual targets found in the active condition can be explained by automatic, exogenous orienting of attention to any appearing sound.

Interestingly, it is possible that in addition to a cross-modal spread of attention, mechanisms related to multisensory integration were also engaged. That is, faster responses on spatially congruent trials could be partly explained by a stronger integration of visual and auditory stimuli. Attention and multisensory integration are thought to be closely linked (Talsma et al., 2010), and a large number of studies have demonstrated stronger shifts of attention toward congruent, integrated compared with incongruent, separated multisensory input (Diederich and Colonius, 2004; Gondan et al., 2005). Irrespectively, if the cross-modal spatial processing bias of a continuous sound could indeed be explained by a series of individual exogenous shifts of attention or mechanisms related to multisensory integration, then the same holds for real-world sounds such as speech, music, or traffic noise. In either case, our present results do allow valid conclusions about the corresponding behavioral effect.

Given the involvement of both attention and cross-modal processing in our task, it is further interesting to consider the present results within the established larger respective theory of Talsma et al. (2010). In their framework, the nature and direction of impact between attention and cross-modal processing are suggested to be largely determined by the complexity and salience of the stimuli, as well as the overall cognitive load. When stimuli in one modality are rare and highly salient (such as in most previous studies on cross-modal attention, e.g., Spence and Driver, 1997; McDonald et al., 2000; Green and McDonald, 2006; Kean and Crawford, 2008), they easily capture bottom-up attention, leading to cross-modal effects on spatially and/or temporally close stimuli in other modalities. If, however, stimuli are of lower saliency (e.g., because they consist of a continuous stream, as in our present experiment), bottom-up effects are reduced, and top-down attention is required for cross-modal processing. Although our data demonstrate a cross-modal impact even during the passive listening condition, indeed, effect sizes for this impact are almost twice as large in the active listening condition (*η*_p_^2^ of 0.27 vs 0.50, respectively). This fits well with the theory by Talsma et al. (2010) but also shows that even under circumstances of low stimulus complexity (such as auditory tone pips) and minimal top-down attention (during the passive listening condition), somewhat smaller cross-modal effects can be observable. In keeping with the framework of bidirectional interplay between attention and cross-modal processing, the underlying neural correlates of our effect might be an increase or a decrease in sensory gain of the rare, task-relevant visual input, depending on whether the (attended) auditory stimulus is spatially congruent or not (Talsma et al., 2010). Several previous studies have demonstrated such attention-mediated, cross-modal neural effects, showing a modification of event-related EEG potentials as early as 80 ms after stimulus presentation (Eimer and Schröger, 1998; Teder-Sälejärvi et al., 1999; Zhang et al., 2011).

We nevertheless take note of several limitations in our study. Ideally, for an optimal comparison between the passive and active listening conditions, the physical stimulation in both conditions should be identical. However, owing to its very nature, the passive lateralized listening condition required unilateral stimulation, whereas the active selective spatial listening condition required bilateral auditory streams (one to selectively attend and one to ignore). Relatedly, it is possible that participants in Experiment 1 partially directed their endogenous attention to the sounds, even if they were task irrelevant, because their auditory attention was not engaged in any other specific task. However, regardless of whether and to what degree this might have been the case, it does not alter our conclusion that continuous, task-irrelevant auditory input can bias the processing of task relevant, spatially close visual stimuli. Finally, it is admittedly difficult to compare the present levels of semantic complexity or task difficulty in the three auditory conditions in Experiment 2. We are also not aware of a straightforward way to control for or equate the physical spectrotemporal properties of such diverse, dynamic sounds as sinewave tone pips and natural speech. Importantly, we observe behavioral differences between semantic complexity conditions only during the active and not passive listening condition. We, thus, argue that it is highly unlikely that these differences are due to low-level physical stimulus properties (e.g., loudness or frequency content), which should have affected performance in a bottom-up way, for instance, by varying individual vigilance levels also during passive listening (Kusnir et al., 2011). Rather, we argue that our observed effects are due to their high-level differences in semantic complexity, which require attentive processing and, thus, were only present in the active listening condition. Relatedly, the scoring of the auditory performance in the HSC condition (comprehension questionnaire about the narrated story) is somewhat more subjective than for the performance in the LSC and MSC conditions (target count). Future studies might employ multiple-choice items rather than open questions to avoid this issue.

## Conclusion

Our study presents several important novel results. We provide the first evidence that continuously attending to an ongoing auditory input facilitates the processing of spatially close visual stimuli. Further, we demonstrate that this bias is present not only during active listening but also when listeners are instructed to ignore the task-irrelevant auditory input. Our results also support previous reports that the semantic complexity of an auditory input impacts the performance in a parallel visual task. Together, our findings demonstrate the implications of ongoing sounds in everyday scenarios such as moving about in traffic, as well as in any profession requiring sustained visual-spatial attention.

## Data Availability Statement

The data are available upon request from the first author.

## Ethics Statement

Ethical review and approval was not required for the study on human participants in accordance with the local legislation and institutional requirements. The patients/participants provided their written informed consent to participate in this study.

## Author Contributions

UP designed the study, conducted the research, analyzed the data, and wrote the manuscript. RS conducted the research and wrote the manuscript. UA designed the study and wrote the manuscript.

## Conflict of Interest

The authors declare that the research was conducted in the absence of any commercial or financial relationships that could be construed as a potential conflict of interest.
