# Supplementary material for: Continuous, Lateralized Auditory Stimulation Biases Visual Spatial Processing
Source: Front Psychol. 2020 Jun 12;11:1183. doi: 10.3389/fpsyg.2020.01183 (PMC7325992; doi:10.3389/fpsyg.2020.01183)
Supplement: Supplementary file 1 [file Presentation_1.pdf]

Supplementary Material to Frontiers Manuscript ID 527078

Gaze fixation analysis and results

In order to investigate potential confounding factors of gaze-fixation behaviour, we first calculated mean fixation times across all subjects, separately for each condition.

Figure S1 shows heatmaps of these data for Experiment 1, with the low-semantic complexity (LSC) condition left and the high-semantic complexity (HSC) right. The top row shows the sound-left condition, the middle row the sound-right condition, and the bottom row shows histograms of fixation times overlaid for both sound-left and sound-right conditions.

To statistically compare the horizontal fixation behaviour between conditions, we computed a repeated measures analysis of variance (ANOVA), with the variables Sound Location (left vs. right) and Semantic Complexity (low vs. high). We found only a trend toward significance for the variable Complexity, $F(1, 16) = 4.17$, $p < .058$, due to a slightly more left average fixation position in the LSC (pixel 636.8) compared to the HSC (pixel 639.9). Importantly however, we observed no effect of Sound Location ($p = 0.67$) and no interaction ($p = 0.45$).

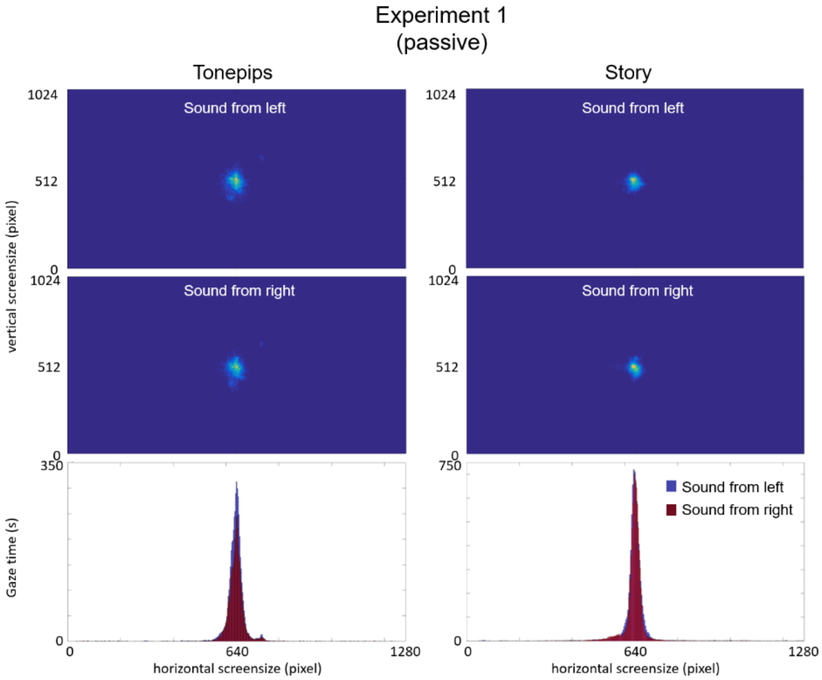

Figure S1.

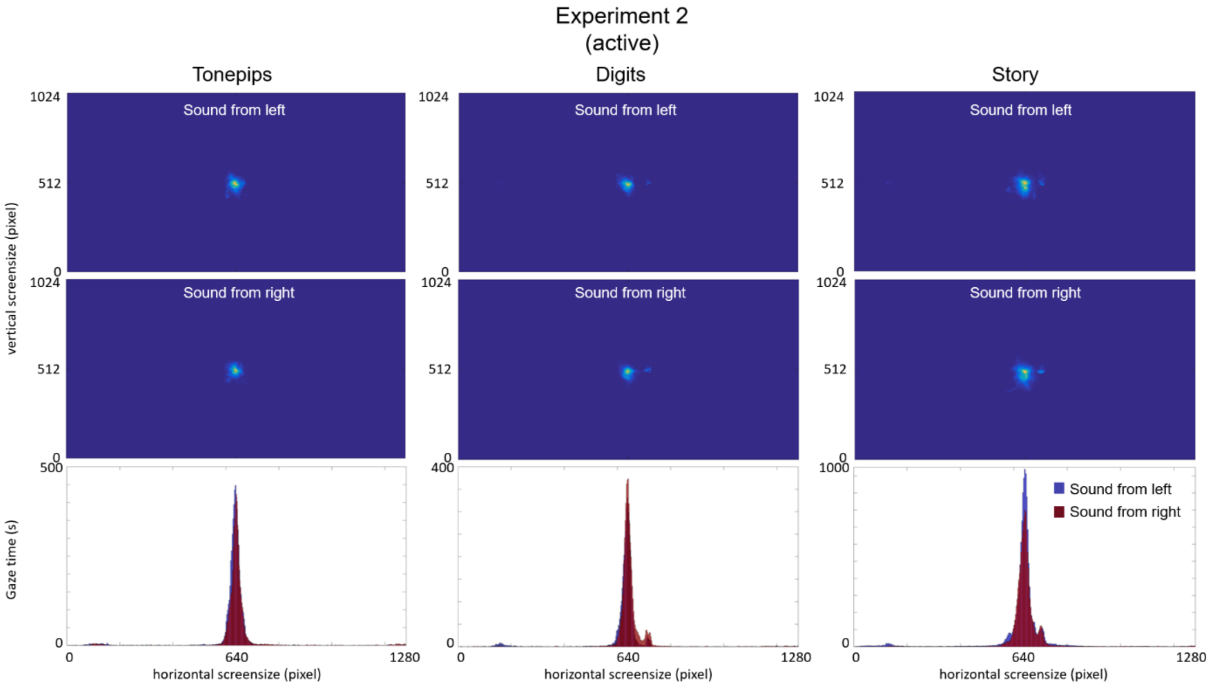

Figure S2.

Figure S2 shows heatmaps of these data for Experiment 2, with the LSC condition left, the MSC condition in the middle, and the HSC condition to the right. The top row shows the sound-left condition, the middle row the sound-right condition, and the bottom row shows histograms of fixation times overlaid for both sound-left and sound-right conditions.

As for Experiment 1, we computed a repeated measures ANOVA with the variables Sound Location (attend-left vs. attend-right) and Semantic Complexity (low vs. med. vs. high) for horizontal fixation behaviour. We found a marginally significant effect of Sound Location, $F(1, 18) = 4.70$, $p < .046$, due to a slightly more left average fixation position in the attend-left (pixel 631.4) compared to the attend-right condition (pixel 641.7). Neither the variable Complexity ($p = 0.73$) nor the interaction ($p = 0.52$) reached significance.

To summarize, only in Experiment 2, we found marginally significant differences between the average fixation locations. Participants fixated 10.3 pixels more to the left during attend-left conditions, compared to attend-right conditions. Due to the small size of this effect, both statistically and in terms of absolute location, we do not consider it to be a confounding factor to our behavioural results.
